# Editorial: New horizons in stroke management

**DOI:** 10.3389/fnhum.2025.1587791

**Published:** 2025-03-28

**Authors:** Nasser Kashou

**Affiliations:** Kash Global Tech, Columbus, OH, United States

**Keywords:** stroke management, biomarkers, post-stroke spasticity (PSS), brain-computer interfaces (BCI), functional electrical stimulation (FES), cognitive impairment (CI), transcutaneous auricular vagus nerve stimulation (taVNS)

## Introduction

Stroke remains one of the leading causes of disability and mortality worldwide, underscoring the urgent need for effective interventions and rehabilitation strategies. Over the years, advancements in the understanding of stroke pathophysiology and rehabilitation techniques have opened new possibilities for improving outcomes for stroke survivors. From novel biomarker discoveries to innovative therapies, new horizons in stroke management are expanding the scope of treatment and recovery. This Research Topic explores several groundbreaking approaches, addressing both acute and long-term consequences of stroke, as well as rehabilitation methods aimed at improving functional recovery.

In the acute phase of ischemic stroke, understanding the biological markers that can predict outcomes is crucial for enhancing patient care. This study focuses on the role of low molecular mass protein 7 (LMMP7) in peripheral blood mononuclear cells as a potential biomarker for stroke severity and prognosis. By analyzing changes in LMMP7 levels from admission to discharge, the study seeks to determine its correlation with disability, recurrence, and mortality, providing valuable insights into prognostic factors that could guide early intervention and management strategies.

LMP7 levels in PBMCs are positively correlated with Th17 cells, inflammation, and the severity of AIS. Importantly, LMP7 levels at discharge are a reliable predictor of stroke recurrence and mortality, making it a potential biomarker for assessing AIS prognosis (Hou and Zhang). Potentially, this marker could be used in clinical practice to help identify high-risk patients and guide treatment strategies.

Post-stroke spasticity can significantly hinder rehabilitation and quality of life for stroke survivors. This article explores the combined use of botulinum toxin A and extracorporeal shockwave therapy (ESWT) as a treatment modality for managing spasticity following stroke. By conducting a systematic review of available studies, the article assesses the efficacy of this combination therapy, evaluating its impact on muscle tone, functional outcomes, and patient satisfaction. This approach may represent a promising intervention for improving motor control and enhancing rehabilitation progress.

Du et al. review the effectiveness of combining botulinum toxin A (BTX-A) with extracorporeal shockwave therapy (ESWT) for treating post-stroke spasticity, based on recent randomized clinical trials. This combination therapy could become a key treatment option for post-stroke spasticity, providing patients with improved mobility, reduced pain, and better overall function. With additional research, this approach may be integrated more widely into clinical practice, offering a non-invasive solution to enhance post-stroke rehabilitation outcomes.

Functional recovery of the upper limb after stroke remains one of the most challenging aspects of rehabilitation. Brain-computer interfaces (BCIs) paired with functional electrical stimulation (FES) represent an innovative way to bridge the gap between neurological deficits and motor recovery. By synthesizing evidence from various clinical trials, the research assesses whether this cutting-edge technology can enhance functional outcomes and speed up recovery for stroke patients with motor impairments.

Ren et al. evaluate the effectiveness of brain-computer interface (BCI)-controlled functional electrical stimulation (FES) training on upper limb functional recovery in stroke patients. A systematic search was conducted across various databases, and randomized controlled trials (RCTs) were included in the analysis. The methodological quality was assessed using the PEDro scale, and a meta-analysis was performed with 290 patients from 10 RCTs.

BCI-FES has significant potential as a rehabilitation tool for improving upper limb function in stroke patients. With further research into its long-term impact and optimal training strategies, BCI-FES could become a widely used method in clinical practice for enhancing motor recovery after stroke. Additionally, incorporating mental tasks like action observation could further optimize rehabilitation outcomes.

Stroke rehabilitation is traditionally approached with a focus either on motor function or cognitive-linguistic abilities, but recent evidence suggests that combining both types of interventions may offer synergistic benefits (Saber-Moghadam et al.). This article delves into the effectiveness of combining motor rehabilitation with language therapy in improving overall recovery outcomes. By exploring how integrated interventions can enhance neuroplasticity and promote a more holistic recovery, this study offers a promising paradigm for rehabilitation that addresses the multi-faceted nature of stroke recovery.

The findings suggest that a combined approach to motor and language rehabilitation could become a more effective strategy in stroke recovery. By targeting both cognitive and physical rehabilitation simultaneously, patients may experience faster and more comprehensive recovery. This integrated approach could be implemented widely in clinical practice, offering a more holistic rehabilitation strategy and potentially improving outcomes for a broader range of stroke patients.

Cognitive impairment is a common and often debilitating consequence of stroke. Traditional rehabilitation methods may be insufficient in addressing long-term cognitive deficits. This case report investigates the use of transcutaneous auricular vagus nerve stimulation (taVNS) as a potential treatment for post-stroke cognitive impairment. By applying diffusion tensor imaging (DTI) to assess brain connectivity and structural changes, the study evaluates the impact of taVNS on cognitive function, offering insights into this non-invasive neuromodulation technique as a promising intervention for long-term stroke survivors (Chen et al.).

This case suggests that taVNS could be a promising non-invasive treatment for long-term cognitive impairment following a stroke. If proven effective in larger studies, it could become a valuable tool for home-based rehabilitation, providing stroke survivors with a feasible and accessible treatment option to enhance cognitive recovery, improve quality of life, and reduce dependency.

## Conclusion

The evolving landscape of stroke management showcases a dynamic approach to both acute care and rehabilitation, with a focus on personalized, evidence-based strategies. From molecular biomarkers and innovative therapies to integrative rehabilitation techniques, these research studies underscore the potential for significant improvements in stroke recovery. As these novel interventions continue to be refined, they promise to offer new hope for those affected by stroke, leading to more effective treatments and enhanced quality of life for stroke survivors.

